# Sural nerve harvest for infants: integrated with information based on anatomical dissections

**DOI:** 10.3906/sag-2005-225

**Published:** 2021-04-30

**Authors:** Mustafa BÜYÜKMUMCU, Anıl Didem AYDIN KABAKÇI, Duygu AKIN SAYGIN, Mehmet Tuğrul YILMAZ, Muzaffer ŞEKER

**Affiliations:** 1 Department of Anatomy, Faculty of Meram Medicine, University of Necmettin Erbakan, Konya Turkey

**Keywords:** Sural nerve, grafts, peripheral nerve injuries

## Abstract

**Background/aim:**

The aim of the present study was to determine the course and possible variations of the sural nerve with all anatomical details in human fetal cadavers.

**Materials and methods:**

This study was performed on 60 fetal cadavers. Formation type and level of the sural nerve was detected.

**Results:**

According to trimesters, it was determined that the mean transverse and vertical distance between the lowest point of the LM and the SN varied between 1.1 and 2.9 mm and 1.54 and 3.58 mm, respectively. Type 2 was the most common seen type of sural nerve (35.83%). It was determined that the sural nerve was mostly formed at the middle third of the leg (42.5%).

**Conclusion:**

Sural nerve graft with the knowledge of the anatomical details may be used for peripheral nerve reconstruction is required in congenital lesions, such as facial paralysis, obstetric brachial paralysis, and posttraumatic lesions in infants and children.

## 1. Introduction

Although nerve grafting is not frequently performed in infants or children, peripheral nerve reconstruction is required in congenital lesions such as facial paralysis, obstetric brachial paralysis, and posttraumatic lesions [1–3].

The sural nerve (SN) is superficially extended, easily dilated, and ideally calibrated for revascularization of interfascicular graft replacement; moreover, its deficiency does not cause significant problems. Furthermore, SN has a long length and minimal branching and is easy to harvest. In terms of these advantages, it is the most common graft source for peripheral nerve injuries that require nerve graft repair [3–10].

The SN is one of the major nerves that transmits sensations to the posterior and lateral-distal portion of the leg, lateral side of the heel, and little toe [4–5,11]. It comprises a union of the lateral sural cutaneous nerve (LSCN) branch of the common fibular nerve and medial sural cutaneous nerve (MSCN) branch of the tibial nerve on the posterior side of the leg. A few studies suggest that the branch that forms the SN through union with the MSCN is furcated from the common peroneal nerve and is called the LSCN [12–13]; however, a few studies claim that there is a branch called the peroneal communicating branch (PCB) that enables the connection between the MSCN and the common peroneal nerve and that this branch may be furcated from the LSCN or directly from the common peroneal nerve [5,7–8,14–15]. These nerves are used in a wide range of applications like the SN for diagnosis (biopsy and neural transmission studies) and treatment (nerve graft), whereas the MSCN is used for free sensorial flaps [13,16]. Moreover, if an additional graft is required, the LSCN can be used [3].

The aim of the present study was to determine the course and possible variations of SN with all anatomical details in human fetal cadavers to remove successful nerve grafts and minimize the risk of SN damage and donor-site scarring during surgical approaches. 

## 2. Materials and methods

The present study was performed on 60 aborted fetal cadavers (34 males, 26 females) without anomaly. The ages of the fetal cadavers were determined to be between the 12th and 33rd postmenstrual weeks based on crown-rump length (CRL) measurements [17]. The fetal cadavers were grouped into first (gestational ages compatible with 12 and 13 weeks), second (gestational ages compatible with 14 and 26 weeks), and third (gestational ages compatible with 27 and 33 weeks) trimesters according to gestational ages. The fetal cadavers were fixed using the immersion technique in 10% formalin. The permission required for the study was obtained in accordance with decision 2012/74 of the Clinical Researches Ethical Committee of Necmettin Erbakan University.

**Figure 1:**
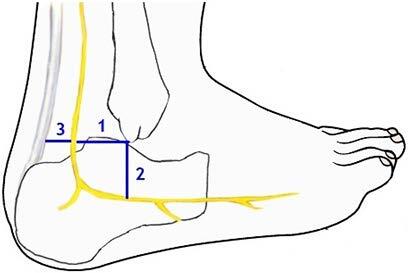
The distance between SN, TC, and LM (1: The transverse distance between SN and LM, 2: The vertical distance between SN and LM, 3: The transverse distance between SN and TC at the lowest point of the LM).

Three incisions were made to the leg. The first incision was applied along a vertical line from the lateral edge of the popliteal fossa toward the tendo calcaneus (TC) on each leg. The second and third incisions were performed transversely at the popliteal fossa and lateral malleolus (LM) levels. Dissection was performed under a surgical microscope (Karl Kaps Sam 62, Germany). Thereafter, formation type and level of the SN was detected on the posterior side of the leg and lateral side of the foot. Measurements (sural nerve length (SNL); the distance between the origin of the SN and the lowest point of the LM, the SN width at the level of the ankle (SNW); the transverse distance (SNW1) between SN and LM, the vertical distance (SNW2) between SN and LM, the transverse distance (SN-TC) between SN and TC at the lower point of the LM) were taken with a digital caliper in the normal position of the foot (Figure 1). Possible variations were photographed. Furthermore, any possible connection branch of the MSCN and LSCN was detected.

In the present study, the formation level of the SN was examined under six groups: 1. no union, 2. union in the popliteal fossa, 3. union in the proximal one-third of the leg, 4. union in the middle one-third of the leg, 5. union in the distal one-third of the leg, 6. union in the ankle.

**Figure 2:**
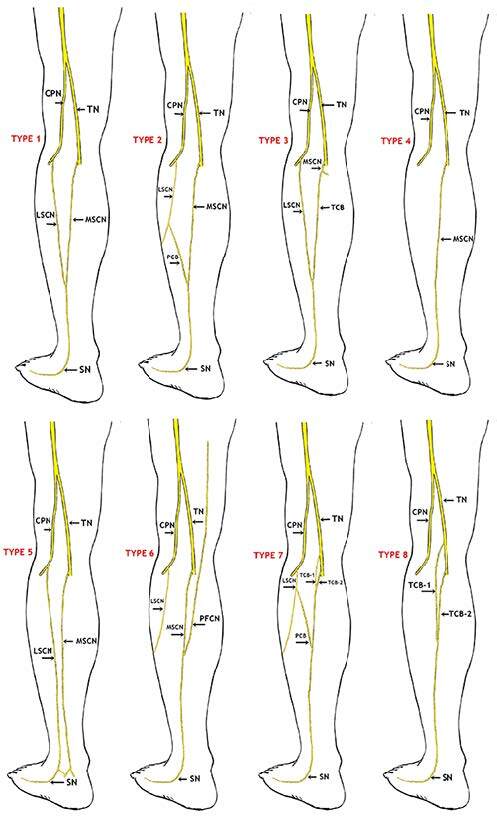
Schematic drawings for sural nerve formations (Type 1–8) (CPN: Common peroneal nerve, TN: Tibial nerve, LSCN: Lateral sural cutaneous nerve, MSCN: Medial sural cutaneous nerve, PCB: Peroneal communicating branch, TCB: Tibial connection branch, SN: Sural nerve, PFCN: Posterior femoral cutaneous nerve).

The SN formations were categorized into eight groups (Figure 2): Type 1: The SN constituted by the union of the MSCN emerging from the tibial nerve and the LSCN emerging from the common peroneal nerve; Type 2: The SN constituted by the MSCN and peroneal connection branch (PCB) emerging from the LSCN; Type 3: The SN constituted by the tibial connection branch (TCB) emerging from the MSCN and LSCN; Type 4: The SN continued as an extension of the MSCN; Type 5: The SN continued as an extension of the LSCN; Type 6: The SN constituted of the MSCN and posterior femoral cutaneous nerve (PFCN); Type 7: The SN constituted of two connection branches (TCB1 and TCB2) emerging from the tibial nerve and connection branch emerging from the LSCN; Type 8: The SN constituted of two connection branches (TCB1 and TCB2) emerging from the tibial nerve.

## 3. Results

According to the trimesters, it was determined that the mean transverse and vertical distance between the lowest point of the LM and the SN varied between 1.1 and 2.9 mm and 1.54 and 3.58 mm, respectively. The mean SN lengths were found to be 13.47 ± 5.05 mm in the first trimester, 24.24 ± 15.39 mm in the second trimester, and 35.44 ± 22.18 mm in the third trimester. The mean SN widths were found to be 0.21 ± 0.04 mm, 0.55 ± 0.21 mm, and 0.68 ± 0.19 mm, respectively (Table 1).

**Figure 3:**
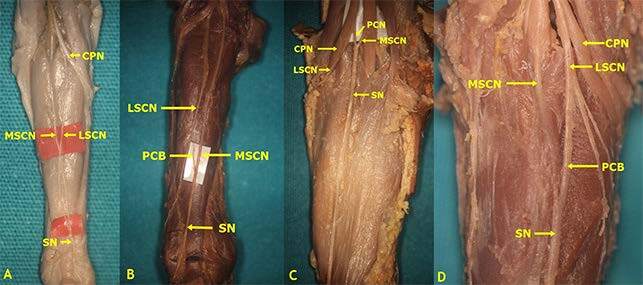
Type 1 and 2 formations of sural nerve (The SN was formed by the PCB from the LSCN joining the MSCN (A: Type 1 - On the right side, the SN was formed by the LSCN from the CPN joining the MSCN at the level of ankle in male fetal cadaver belonging to 2nd trimester; B: Type 2 - On the left side, at the middle third of the leg in male fetal cadaver belonging to 3rd trimester; C: Type 2 - On the left side, at the popliteal fossa in male fetal cadaver belonging to 3rd trimester; D: Type 2 - On the right side, at proximal third of the leg in female fetal cadaver belonging to 2nd trimester) (CPN: Common peroneal nerve, LSCN: Lateral sural cutaneous nerve, MSCN: Medial sural cutaneous nerve, PCB: Peroneal communicating branch, SN: Sural nerve).

**Figure 4:**
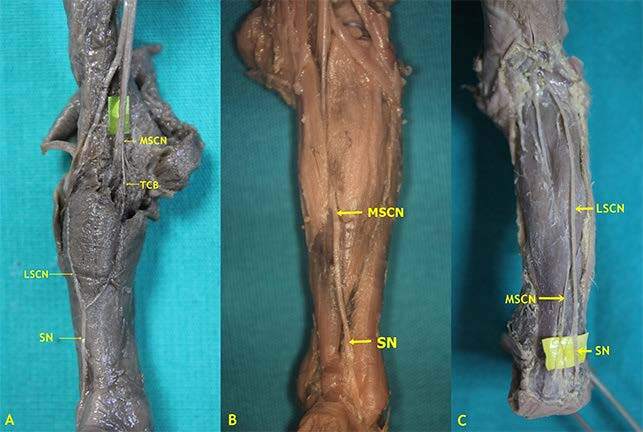
Type 3, 4, and 5 formations of sural nerve in male fetal cadaver belonging to 2nd trimester (A: Type 3- On the left side, the SN was formed by the TCB from the MSCN joining the LSCN separated from the CPN at the middle third of the; B: Type 4-On the left side, the SN was formed by the only MSCN; C: Type 5- On the right side, the SN was formed by the only LSCN) (LSCN: Lateral sural cutaneous nerve, MSCN: Medial sural cutaneous nerve, TCB: Tibial connection branch, SN: Sural nerve).

**Figure 5:**
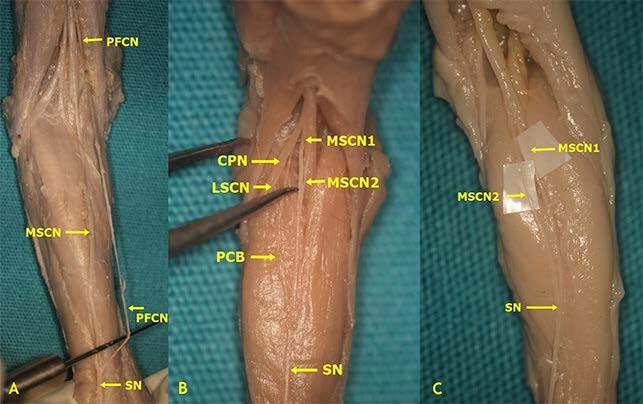
Type 6, 7, and 8 formations of sural nerve (A: Type 6-On the left side, the SN was formed by posterior femoral cutaneous nerve joining the MSCN at the level of ankle in female fetal cadaver belonging to 2nd trimester; B: Type 7-On the left side, the SN was formed by the PCB from the LSCN joining the MSCN that consisted of merging two branches separated from TN at the middle third of the leg in male fetal cadaver belonging to 1st trimester; C: Type 8-On the left side, the SN was formed by the MSCN that consisted of merging two branches separated from TN at the middle third of the leg in male fetal cadaver belonging to 1st trimester) (CPN: Common peroneal nerve, LSCN: Lateral sural cutaneous nerve, MSCN: Medial sural cutaneous nerve, PCB: Peroneal communicating branch, SN: Sural nerve, PFCN: Posterior femoral cutaneous nerve).

In the present study, Type 1 was detected in 21 (35%) left lower limbs and 20 (33.3%) right lower limbs. Type 2 was detected in 20 (33.3%) lower limbs on the left and 23 (38.3%) lower limbs on the right (Table 2, Figures 2, 3A–3D). Type 3 was detected in one limb on the left only whereas it was not detected in any of the right limbs (Table 2, Figures 2 and 4A). Furthermore, it was detected that the SN courses as a continuation of the MSCN (Type 4) in 25 (20.83%) of the 120 lower limbs and a continuation of the LSCN in 5 of the lower limbs (4.17%) (Type 5) (Table 2, Figures 2, 4B, and 4C). Type 6 was detected in 1 (0.83%) lower limb only (Table 2, Figures 2 and 5A). Furthermore, Type 7 was detected in 2.5% of the cases on the left and right lower limbs, whereas Type 8 was detected in 0.83% of all lower limbs (Table 2, Figures 2, 5B, and 5C).

**Table 1:** The mean values of SN and neighboring structures (N: number of samples, mm).

PARAMETERS	TRIMESTER					
	1st trimester (n=10)		2nd trimester (n=29)		3rd trimester (n=21)	
	Gestational weeks (12–13)		Gestational weeks (14–26)		Gestational weeks (28–33)	
	Mean	Std. deviation	Mean	Std. deviation	Mean	Std. deviation
SNL	13.47	5.05	24.24	15.39	35.44	22.18
SN width	0.21	0.04	0.55	0.21	0.68	0.19
SNW1	1.1	0.4	1.9	0.8	2.9	0.9
SNW2	1.54	0.55	2.29	0.91	3.58	1.17
SN-TC	0.69	0.25	1.34	0.59	2.55	1.08

SN: Sural nerve, SNL: Sural nerve length, SN width: sural nerve width at the level of ankle, SNW1: The transverse distance between sural nerve and lateral malleolus, SNW2: The vertical distance between sural nerve and lateral malleolus, SN-TC: The transverse distance between sural nerve and tendo calcaneus at the lower point of the lateral malleolus.

**Table 2:** The formation types of sural nerve (%).

	Left			Right		
	Male	Female	Total	Male	Female	Total
Type 1	12(35.3%)	9(34.6%)	21(35%)	12(35.3%)	8(30.8%)	20(33.3%)
Type 2	11(32.4%)	9(34.6%)	20(33.3%)	12(35.3%)	11(42.3%)	23(38.3%)
Type 3	1(2.9%)	0(0%)	1(1.7%)	0(0%)	0(0%)	0(0%)
Type 4	6(17.6%)	6(23.1%)	12(20%)	7(20.6%)	6(23.1%)	13(21.7%)
Type 5	2(5.9%)	0(0%)	2(3.3%)	3(8.8%)	0(0%)	3(5%)
Type 6	0(0%)	1(3.8%)	1(1.7%)	0(0%)	0(0%)	0(0%)
Type 7	1(2.9%)	1(3.8%)	2(3.3%)	0(0%)	1(3.8%)	1(1.7%)
Type 8	1(2.9%)	0(0%)	1(1.7%)	0(0%)	0(0%)	0(0%)
Total	34(100%)	26(100%)	60(100%)	34(100%)	26(100%)	60(100%)

The SN formation levels were detected in the following manner: no union (24.2%); union on popliteal fossa (3.3%); union on proximal one-third of the leg (0.8%); union on middle one-third of the leg (42.5%); union on distal one-third of the leg (22.5%); and union on the ankle (6.7%). Furthermore, the present study determined that the MSCN emerged most from the posterior side of the tibial nerve (90 legs: 75%), while the LSCN emerged most from the posterolateral side of the common peroneal nerve (102 legs: 85%).

## 4. Discussion

The SN is the most commonly used donor nerve for nerve grafts in peripheral nerve reconstruction surgery. The SN is used in interfascicular graft replacement to alter the damaged nerves in cases such as facial paralysis, obstetric brachial paralysis, and post-traumatic lesions [2]. The damaged nerves need to be repaired in time. The best time for these operations is the first year of life. In particular, the first 3 months of life are the ideal age for nerve repair in severe injuries [18]. The SN harvest in adults is easier than in infants and children because the relationship between SN and related structures is better known among the former. It is known that SN is located approximately 1.5 cm posterior and almost 1 cm to the superior point of the LM [10]. Based on our dissection study and the measurements of fetal cadavers belonging to the third trimester, important reference points were obtained for the SN harvest in infants by considering the distances between the SN and LM. The mean transverse and vertical distance between the lowest point of the LM and the SN were found to be 2.9 ± 0.9 mm; 3.58 ± 1.17 mm, respectively, for the third trimester.

Recently, there has been an increase in the utilization of SN grafts in the treatment of peripheral nerve injuries, like obstetrical brachial plexus palsy in children. Having detailed anatomical knowledge of SN and its related bone structures is important during the nerve harvest process. The conventional techniques used for SN harvest are performed with a single longitudinal incision or multiple “stair-step” incisions. This enables direct visualization of the nerve and minimizes nerve injury. Scars may have a long, numerous and poor appearance in conventional techniques. Certain researchers have suggested that small skin incisions must be made, and a stripper device must be used to minimize the negative effects of traditional methods in the SN harvest. Although nerve harvesting with the stripper device is possible quickly and has significant advantages in reducing scarring, there is a possibility of injury to the SN and lesser saphenous vein, as the harvest is done blindly [19–21]. For this reason, endoscopy-guided nerve harvesting enables direct monitoring of the nerve and minimizes the risk of injury to other structures in the region. Irrespective of the method used—traditional methods, stripper device, or endoscopy-guided nerve harvesting—the differences in SN formation depending on the branching patterns of the MSCN and LSCN and a peroneal communicating branch may affect the SN harvest.

Further, branching patterns of the SN were indicated in fetal, neonatal, and adult cadavers of different populations or in radiological studies from the first year of identification to date [4–9,11,13–16,22–28].

According to our results, Type 1 formation was detected in 41 (34.17%) lower limbs (Figures 2 and 3A). In consideration of SN formation, several studies performed on fetal cadavers in the literature revealed different rates: 67.5% in the study by Uluutku et al. [15] on 40 legs of 20 fetuses; 9% in the study by Ugrenovic et al. [25] conducted on 200 lower limbs of 100 fetuses (18 lower limbs); 68% in the study by Albay et al. [8] on 100 lower limbs of 50 fetuses (68 lower limbs); and 12.5% in the study of Reis et al. [9] on 40 lower limbs of 20 fetuses. Furthermore, Type 2 formation, which is formed by union of the MSCN and the peroneal communicating branch emerging from the LSCN, was detected on 43 (35.83%) lower limbs (Figures 2, 3B–3D) in our study. Such rates were detected as 10% (4 legs) in the study by Uluutku et al. [15] conducted on 40 legs of 20 fetuses; 58.5% in the study by Ugrenovic et al. [25] conducted on 200 lower limbs of 100 fetuses (117 lower limbs); 3% in the study by Albay et al. [8] conducted on 100 lower limbs of 50 fetuses (3 lower limbs). The incidence of Types 1 and 3 together, which was detected by Reis et al. [9] in 40 lower limbs of 20 fetuses is similar to our incidence rates in Type 2; however, they detected this rate as 42.5%. Furthermore, Desdicioğlu et al. [28] classified Types 1 and 2 as anastomotic type and found the rate to be 69.56%. This rate is consistent with the data in our study.

In the present study, Type 3 formation, which appears by the union of the connection branch (TCB) emerging from the MSCN and LSCN, was detected only in one lower limb (1.7%) (Figures 2 and 4A). No previous study has reported that the tibial communicating branch was separated from the tibial nerve. Type 4 formation as a continuation of the MSCN was detected in 25 (20.83%) of 120 lower limbs (Figures 2 and 4B). This rate was found to be 12.5% by Uluutku et al. [15], 26.5% by Shankar et al. [7], 26% by Ugrenovic et al. [25], 20% by Albay et al. [8], 10% by Reis et al. [9], 19.56% by Desdicioğlu et al. [28], and 8% by Ulcay and Uzun [6]. Type 5 formation coursing as a continuation of the LSCN was detected on 5 (4.17%) of 120 lower limbs (Figures 2 and 4C). This rate in fetal cadavers was found to be 1.5% by Ugrenovic et al. [25], 22.5% by Shankar et al. [7], 1% by Albay et al. [8], 6.52% by Desdicioğlu et al. [28], and 0% by Ulcay and Uzun [6]. Type 6 formation appearing due to the union of the MSCN and PFCN was detected in 1 (0.83%) of 120 lower limbs (Figures 2 and 5A). There are studies that support the participation of the nerve fiber from the PFCN in SN formation. Uluutku et al. [15] reported that SN formation involved the PFCN with peroneal connection and MSCN in 2 (5%) of 40 lower limbs. Desdicioğlu et al. [28] found Type D formation through a combination of different nerves such as the MSCN, LSCN, PFCN, and the sciatic nerve (ScN) to be 4.36%. Ugrenovic et al. [25] reported one (0.5%) case only. Among the cases identified as Type A by Shankar et al. [7] where the union of the MSCN and peroneal communicating branch (PCB) forms the SN, fibers from the PFCN were detected in three (7.9%) limbs only. Albay et al. [8] determined this form as Type C and the rate as 4%. Eid and Hegazy [11] performed a study on the lower limbs of 24 adult cadavers and reported the rate of the cases where the MSCN, PCB, and PFCN together form the SN at a rate of 12.5% (three lower limbs). Furthermore, Type 7 formation, which appears from the union of two connection branches emerging from the tibial nerve and PCB that emerge from the LSCN, was detected in 3 (2.5%) of 120 lower limbs (Figure 2 and 5B). Type 8, which appears from the union of 2 communicating branches that emerge from the tibial nerve, was detected in 1 (0.83%) of 120 lower limbs (Figure 2 and Figure 5C). 

Nerve grafts must be 10–15% greater than the actual measured length [29]. In particular, if additional length is needed for the graft, the lateral sural nerve (a PCB) can be used for harvesting [3]. If an additional graft is used, it is important to know whether the sural formation is anastomotic or nonanastomotic. A few researchers categorized SN formation under three groups: anastomotic (Type A) type, nonanastomotic tibial type (Type B), and nonanastomotic peroneal type (Type C). In our study, the anastomotic type, among Types 1, 2, 3, and 7, was observed in 88 out of 120 legs (73.33%). The tibial nonanastomotic type (Types 4 and 8 in our study) was observed in 26 out of 120 (21.66%) legs. The peroneal nonanastomotic type (Type 5 in our study) was observed in 5 legs (4.16%) (Table 3).

**Table 3:** The percentage of the formation of the sural nerve according to the researchers (%).

Researchers	Samples	Anastomotictype (Type A)	Nonanastomotictibial type (Type B)	Nonanastomoticperoneal type (Type C)
Catania [22]	100 lower extremities	51.0%	35.0%	14.0%
Ortigiiela et al. [14]	20 lower extremities	80%	20%	-
Mestdgah et al. [23]	37 lower extremities	67.57% (25 cases)	24.32% (9 cases)	8.11% (3 cases)
Uluutku et al. [15]	20 new-born cadavers (40 legs)	77.5%	12.5%	-
Mahakkanukrauh andChomsung [13]	152 lower extremities	67.1%	32.2%	-
Ugrenovic et al. [25]	100 human fetuses	67.5%	26%	6.5%
Aktan İkiz et al. [26]	30 lower extremities	70% (18+3 cases)	23.33% (5+2 cases)	6.67% (2 cases)
Sekiya et al. (2006)	31 lower extremities	74.2% (23 cases)	22.6% (7 cases)	3.2% (1 case)
Shankar et al. [24]	51 fetuses	37.2%	26.5%	22.5%
Zhu et al. [27]	100 ultrasonographicimages of patients	81%	18%	1%
Eid and Hegazy [11]	24 lower extremities	87.5%	12.5%	-
Albay et al. [8]	50 embalmed fetuses	71%	20%	%1
Kavyashree et al. [4]	50 lower extremities	72% (36 cases)	28% (14 cases)	-
Seema [5]	100 lower extremities	60%	39%	1%
Reis et al. [9]	20 human fetuses (40 legs)	55%	10%	-
Desdicioğlu et al. [28]	46 lower extremities of 23 fetuses	69.56%	19.56%	6.52%
Ulcay and Uzun [6]	18 stillborns	92%	8%	0%
Our study	60 abort fetal cadavers(34 males, 26 females)	73.33%	21.66%	4.16%

In the table, sural nerve were classified under three categories. The different classifications and rates used by some researchers in their studies are not included.

Traditionally, “stocking seam” or “stair-step” incisions were used for SN harvesting [19]. Recently, these methods have not been preferred for use on account of unsightly scars, increase in operating time, insufficient number of grafts harvested, and possibility of equinovarus deformities [30]. Of late, SN harvesting is performed with fewer incisions. In almost all techniques, the first incision is at the level of the LM. If needed, the second incision must be performed at the most appropriate place to both harvest the maximum length of nerve grafts and reduce scarring. Furthermore, knowing the formation site of the SN may be useful in cases where two or more incisions are required.

Studies using adult cadavers have revealed that the formation level is also in the distal and middle one-third of the leg. Mahakkanukrauh and Chomsung [13] conducted a study on 76 cadavers and found a formation level of 5.9% in the popliteal fossa, 1.9% in the middle one-third of the leg, 67.4% in the distal one-third of the leg, and 25.5% in or below the ankle. Mestdagh et al. [23] reported a formation between the proximal two-third and distal one-third of the leg (57%, which appeared approximately 2 mm below the transverse fold of the popliteal fossa). Pyun and Kwon [16] stated that the connection between the MSCN and LSCN appears in the middle one-third of the leg in 9 (45%) cases and on the distal one-third of the leg in 11 (55%) cases. Eid and Hegazy [11] emphasized the most common formation site of the SN in the distal one-third of the leg and ankle (62%). Similarly, Kavyashree et al. [4] found the common formation site of the nerve in the distal one-third of the leg (58.3%). 

Uluutku et al. [15] stated that formation is mostly in the middle one-third (27–81.8%, distal one-third (5%–15.2%), and proximal one-third (1–3%) of the leg. Reis et al. [9] detected the most common formation sites of the SN as the middle one-third of the leg by 54.5%, distal one-third by 36%, and proximal one-third by 9.5%. The outcomes given by Ugrenovic et al. [25] and Albay et al. [8] are in line with those obtained by Uluutku et al. [15] and indicate that the most common formation site of the SN is the middle one-third of the leg. Ulcay and Uzun [6] detected the most common formation site as the third quarter of the leg (70%; 23 legs). Furthermore, Shankar et al. [7] performed a study on 51 fetuses and reported that the most common formation site of the SN is the distal two-thirds of the leg by 95%. Shankar et al. also stated that the SN appears in the middle one-third of the leg in males, whereas it appears in the distal one-third of the leg in females. In our study, the most common formation site was the middle one-third of the leg (42.5%). Unlike other studies, Desdicioğlu et al. [28] found the most common formation site of SN to be the distal one-third of the leg (48%; 22 cases). 

Having knowledge regarding the general anatomical structure of the region and possible variations during the application of surgical procedures accords superiority to a surgeon. In particular, in peripheral nerve injuries, the best time for nerve grafting is the first 3 months after birth. The SN is frequently used for this purpose. In this study, we revealed the anatomical details necessary for SN harvesting, particularly in intrauterine nerve surgery, based on anatomical dissection. We believe that the data obtained from this study will serve as a guide during the replacement of damaged nerves, particularly in younger individuals.

### 4.1. Limitations

This study was conducted on a large population. Important data were obtained in terms of the course of the SN and follow-up variations. The normal course of the sural nerve was determined and nerve variations that can affect the success rates of surgical operations were identified. The most important limitation of the study was measurement on relatively fewer third-trimester fetal cadavers. We believe that the data obtained from the third-trimester and full-term samples will be valuable, particularly in the operations planned in the first 3 months after birth.

## Informed consent

This study conformed to the Helsinki Declaration. We confirm that we have read the Journal’s position on issues involved in ethical publication and affirm that this report is consistent with those guidelines. The permission required for the study was obtained in accordance with decision 2012/74 of the Clinical Researches Ethical Committee of Necmettin Erbakan University.

## Author contributions

All authors have participated in conception and design, or analysis and interpretation of the data; drafting the article or revising it critically for important intellectual content; and approval of the final version.
